# Interorganizational Sensemaking of the Transition Toward a Circular Value Chain

**DOI:** 10.1177/10860266231162057

**Published:** 2023-04-04

**Authors:** Marianne Kuhlmann, Johannes Meuer, Catharina R. Bening

**Affiliations:** 1 ETH Zurich, Switzerland; 2 Kühne Logistics University, Germany

**Keywords:** case studies, ethnographic and action research, circular economies, sensemaking, identity

## Abstract

The transition toward the circular economy requires stakeholders to collaborate along value chains. Yet, such collaborations are considerably challenging. Given the paradigmatic change, stakeholders face high levels of uncertainty and also need to align on a common way forward. We extend research on interorganizational sensemaking and the circular economy by exploring the process of interorganizational alignment in a European consortium of over 150 companies representing the value chain for flexible packaging with the objective to transform the value chain from linear to circular. We find that the interorganizational sensemaking process unfolds across three levels—organization, value chain, and ecosystem—which provide different reference frames for the process. We provide insights into how these frames, power dynamics, and identity considerations influence this process. Our findings highlight the importance of considering interdependencies between stakeholders and a collective reconceptualization of the established value chain to successfully transition toward a circular one.

## Introduction

In the light of the continuously worsening climate crisis, the concept of a circular economy is gaining attention as an alternative to the established economic “take-make-waste” system that has been criticized for leading to the overexploitation of material resources and the destruction of our natural environment (Meadows & Randers, 2012; Stahel, 2016). The transition from a linear to a circular economy is a paradigmatic shift that requires a fundamental restructuring of economic activities along value chains (Korhonen et al., 2018; Ruggieri et al., 2016). Individual actors cannot implement the associated changes alone, as they require the collaboration of other actors whose activities are interdependent with theirs (Brown et al., 2021; Parida et al., 2019). Collaborative initiatives between stakeholders have therefore gained importance as a powerful tool for achieving such concerted action.

Yet, such collaborations often face considerable challenges as the transition toward a circular economy involves considerable uncertainty (Korhonen et al., 2018). The restructuring of economic activities is associated with fundamental changes in production and consumption patterns, including the development of new products and services, processes, technologies, and overall business models (Bocken & Geradts, 2020; Eikelenboom & de Jong, 2022; Tapaninaho & Heikkinen, 2022). More importantly, actors often lack a common understanding of the concept of a circular economy and its ultimate goals (Kirchherr et al., 2017; Moraga et al., 2019). Managers, therefore, face considerable uncertainty when evaluating different potential approaches to applying the circular concept to their business (Bocken & Geradts, 2020; Centobelli et al., 2020; Werning & Spinler, 2020). These challenges are exacerbated by the need to implement changes across the value chain in collaboration with interdependent stakeholders (Bocken & Geradts, 2020; Eikelenboom & de Jong, 2022). Thus, to successfully transition toward a more circular structure, stakeholders must develop a joint understanding of the change and its implications for both individual organizations and the overall industry and, importantly, align on a common way forward, while accommodating potential tensions between individual and collective interests (Eikelenboom & de Jong, 2022; Parida et al., 2019; Tapaninaho & Heikkinen, 2022).

However, as most research has focused on the outcome of such endeavors rather than the interaction between stakeholders, little is known about the dynamic process of interorganizational alignment (Pieroni et al., 2019; Seidl & Werle, 2018; Selsky & Parker, 2005). Thus, researchers have begun to call for immersive studies to reveal the complex social process of circular economy transitions (e.g., Bertassini, Ometto, et al., 2021). We adopt such an approach to help fill this gap. We ask the following question: *How do stakeholders from one value chain develop a joint understanding of the transition toward a circular economy and align on a common way forward?*

To address this question, we adopt the lens of interorganizational sensemaking (Maitlis & Christianson, 2014; Seidl & Werle, 2018; Weick, 1995). Interorganizational sensemaking refers to the processes that unfold between organizations as they collectively engage with each other to make sense of changes in the external environment, thereby constructing a shared meaning that helps them to reduce ambiguity and act accordingly (Maitlis & Christianson, 2014; Seidl & Werle, 2018). The sensemaking lens provides a useful frame for analyzing the alignment process of a group of organizations collaborating to deal with a major change such as the transition toward a circular economy.

Our analysis is based on an in-depth case study of a European consortium of more than 150 companies representing the entire value chain for flexible packaging, with the goal of transforming the value chain from linear to circular. Between September 2019 and December 2020, we supported the consortium to identify topics that stakeholders considered essential for the successful circular transformation but had diverging opinions on and to facilitate the stakeholders’ alignment process to reach a common position. By observing and analyzing these intense negotiations, we show how the complex sensemaking process between organizations unfolds across three levels: organization, value chain, and ecosystem. In addition, we identify five key subprocess steps and shed light on the dynamics that shape them. In doing so, we contribute to the literature on interorganizational sensemaking and provide insights for circular economy research and practice by revealing the interorganizational dynamics of the transformation from a linear to a circular value chain.

The remainder of the article is structured as follows: Section 2 provides the theoretical background, while Section 3 outlines our methodological approach. Section 4 presents our findings, followed by a discussion in Section 5. Section 6 concludes.

## Theoretical Background

### Organizational and Interorganizational Sensemaking

Sensemaking refers to the process through which actors attempt to understand issues, events, or actions that are novel, ambiguous, unexpected, or confusing (Maitlis, 2005; Weick, 1995). There are various triggers for sensemaking, such as external shocks and crises, threats to identity, and also planned organizational interventions (Maitlis & Christianson, 2014; Weick, 1995). Actors perceive these triggers as disrupting their established understanding of their environment and are uncertain how they should act, so they seek to make sense of them. Sensemaking can be considered a social process, during which actors extract and interpret cues from their environment and engage in dialogs to construct a shared meaning that helps them understand the cues and act collectively (Maitlis & Christianson, 2014; Seidl & Werle, 2018; Weick, 1995).

However, sensemaking can be difficult. As a social process, the collaborative endeavor is influenced by differences in the interests and knowledge of the actors involved, as well as social dynamics that may emerge between them. Not only may actors consider different cues important, and hence engage differently in a collaborative sensemaking process (Maitlis, 2005; Schildt et al., 2020; Seidl & Werle, 2018), they may also use different frames for interpretation based on their particular knowledge structure and past experiences. While such diversity is helpful for making sense of complex changes, it can also create tensions, as stakeholders must ultimately align on a certain interpretation schema. Indeed, the collaborative sensemaking process can be subject to power dynamics between actors, as they try to influence its outcome in their own favor (Clegg, 1989; Fleming & Spicer, 2014; Schildt et al., 2020).

Power plays can be open and direct, as actors deliberately try to coerce, influence, or manipulate others (Clegg, 1989; Schildt et al., 2020). Sensebreaking and sensegiving are important aspects of such episodic power play. Individuals may engage in sensebreaking— “the destruction or breaking down of meaning” (Pratt, 2000, p. 464)—to disrupt and invalidate the established understanding and sense of self of others and create a void of meaning that motivates them to search for a new meaning (Pratt, 2000; Schildt et al., 2020). Individuals, and leaders in particular, may take advantage of this void by engaging in sensegiving, that is, the “process of attempting to influence the sensemaking and meaning construction of others toward a preferred redefinition of organizational reality” (Gioia & Chittipeddi, 1991, p. 442) to shape the collective sensemaking process. This can also lead to “framing contests” (Kaplan, 2008) between actors, as they try to make their individually preferred frame dominate collective sensemaking. Sensebreaking is often a precursor to sensegiving, as successful sensebreaking induces seekership for new meaning and, thus, a greater receptiveness to sensegiving (Schildt et al., 2020). However, attempts at sensebreaking and sensegiving may also fail and lead to disidentification from the group as actors reaffirm the established sense or reject the imposed one (Pratt, 2000).

In addition to direct power used by individuals, the structural context also shapes actors’ sensemaking activities through its systemic power. Systemic power refers to established knowledge structures and identity perceptions that shape the way actors see the world and act (Clegg, 1989; Lawrence et al., 2012; Schildt et al., 2020). Similar to episodic power, systemic power can have different effects on the way actors make sense of a situation. It can have a conservative influence and lead to narrow sensemaking in which the broader setting is not questioned, but it can also open up the solution space by “drawing attention to the inadequacy of present actions as plausible solutions to the issues at hand” (Schildt et al., 2020, p. 253). In particular, an increase in diversity of knowledge can lead actors to question prior beliefs and induce a change in evaluation criteria (Schildt et al., 2020). Systemic power can also be instrumentalized by individual actors to alter the structural frame and influence the sensemaking process indirectly (Maitlis, 2005; Schildt et al., 2020).

Actors’ sensemaking is connected to their perception of identity—that is, members’ understanding of “who we are as an organization” (Gioia et al., 2013, p. 123). While previous research has mostly focused on how identity is constructed through sensemaking (Maitlis & Sonenshein, 2010; Sandberg & Tsoukas, 2015), actors’ perception of identity may also influence sensemaking, as who people think they are also shapes how they interpret novel events. According to Schildt et al., (2020, p. 253), “identity is a particularly strong driver for committed sensemaking because there is hardly anything that feels as plausible and certain to actors as their own established identities.” Thus, identity can be considered a conduit of systemic power that influences the sensemaking process. Indeed, external changes may create a conflict with one’s established identity that can become a potent source of doubt and lead to a search for entirely new understandings (Christianson et al., 2009; Schildt et al., 2020). As Weick (1995, p. 23) points out, “intentional sensemaking is triggered by a failure to confirm one’s self.” That is, the contradiction of identity can lead to deeper and broader sensemaking in the active search for renewed coherence. Thus, sensemaking may ultimately also lead to changes in the perception of identity (Christianson et al., 2009; Dutton & Dukerich, 1991; Maitlis & Christianson, 2014). As Weick postulates, “What the situation means is defined by who I become while dealing with it” (Weick, 1995, p. 24). Given the importance of individual and collective perceptions of identity, they may also become subject to powerplay in interorganizational sensemaking, as actors try to influence others’ perceptions of identity and stir up identity conflict (Schildt et al., 2020).

Research on sensemaking has largely focused on sensemaking within organizations. Yet, sensemaking also happens when actors from different organizations engage in interorganizational sensemaking (Seidl & Werle, 2018). The different perspectives of the collaborators increase the diversity of schemata to interpret interrelated aspects and, thus, help actors develop a more comprehensive understanding (Maitlis & Christianson, 2014; Seidl & Werle, 2018; Weick, 1995). So far, however, little research has specifically focused on the interorganizational sensemaking process and the relationship dynamics that may unfold between diverse actors. Seidl and Werle (2018) analyze the interorganizational process as a way for individual actors to access a greater variety of frames in their own sensemaking. However, their study is limited to individual understanding and does not analyze relationship and power dynamics, nor how actors may actually align on a common position. Yet, such dynamics are crucial in the context of interdependent actors who participate in collaborative sensemaking to align on a common way forward. If such interdependencies exist, actors may require not only knowledge and interpretation schemata from others but also a mutually agreed interpretation as a basis for concerted joint action.

Also, little is known about any potential interplay of sensemaking processes at the organizational and industry levels. Cristofaro (2022) suggests that supra-organizational aspects such as the actors’ perception of industry identity influence the sensemaking process on the organizational level. However, potential conflicts between the actors’ interests and perceptions of changing industry dynamics are not addressed. Equally, the research of Stigliani and Elsbach (2018) on identity formation and that of Patvardhan et al. (2015) on a meta-level identity crisis hint at such an interplay of sensemaking at the organizational and industry levels. However, they focus on identity formation rather than the broader sensemaking process; whether and how the interorganizational sensemaking process may unfold across different levels remain underexplored.

Given these limited insights, researchers have called for more studies that explore the interplay of sensemaking across organizations, particularly tensions and dynamics (Maitlis & Christianson, 2014; Seidl & Werle, 2018). Such insights can be especially valuable for the growing number of collaborative endeavors seeking to address complex changes that shake up entire industries—such as the transition toward the circular economy. Such paradigmatic shifts put pressure on established structures, relationships between actors, and ways of doing things and require holistic adaptations. Given the interdependencies of actors along value chains, interorganizational sensemaking of such shifts is essential to align on concerted action. This article aims to offer new insights into this process.

### Collaborating for a Circular Economy

For a circular economy to unfold, economic activities along the value chain must be fundamentally restructured (Bocken et al., 2016; Ruggieri et al., 2016). These changes cannot be realized by individual firms alone but require the collaboration of interdependent stakeholders along the value chain (Brown et al., 2021; Parida et al., 2019). Yet such concerted engagements often face considerable challenges. Stakeholders may hold different conceptualizations of the transition toward a circular economy, as it entails considerable uncertainty and challenges established structures (Eikelenboom & de Jong, 2022; Kirchherr et al., 2018; Korhonen et al., 2018). A common understanding of the concept of a circular economy, including its goals and systems of measurement, is still missing (Kirchherr et al., 2017; Moraga et al., 2019). In addition, a fundamental restructuring of economic activities along the value chain is required to allow for the continuous reuse, recycling, and looping of materials back into the economy. This restructuring is associated with fundamental changes in production and consumption patterns, including the development of new products and services, processes, and technologies, as well as overall business models (Eikelenboom & de Jong, 2022; Tapaninaho & Heikkinen, 2022). Given the relatively recent uptake of the concept in the business realm, the success of the different approaches remains uncertain. Managers, therefore, need to consider many different potential methods for applying the circular concept to their business (Bocken & Geradts, 2020; Centobelli et al., 2020; Werning & Spinler, 2020). Also, because the transition to a circular system centers around altering physical material flows throughout the economy, it is difficult for individual economic actors to understand and evaluate the feasibility of specific solutions and required changes from the production of materials to their recycling, as these often span a broad set of economic activities realized by many different actors whose activities are interdependent (Bertassini, Zanon, et al., 2021; Brown et al., 2021). Thus, to transition from a linear to circular model, managers must actively engage with other stakeholders to interpret what circularity really means and to determine its concrete implications for their organization.

In addition, given the interdependencies along the value chain, stakeholders must also agree on how they should collectively adapt. This can be difficult for individual stakeholders because the required adaptations may interfere with the current linear reality of their business and create conflicts of interest (Eikelenboom & de Jong, 2022). Significant efforts are, therefore, often required to align diverging perspectives and interests of different stakeholders and achieve concerted action (Bening et al., 2021; Bridoux & Stoelhorst, 2022). Accordingly, scholars frequently emphasize the importance of interfirm collaboration (e.g., Bertassini, Zanon, et al., 2021; Bocken & Geradts, 2020). However, while research has started to explicitly focus on the organizational perspective of the transition to a circular economy (Bocken & Geradts, 2020; Brown et al., 2021; Centobelli et al., 2020; Eikelenboom & de Jong, 2022; Pieroni et al., 2019), studies have so far mostly focused on the organizational dynamics of individual firms; the interorganizational dynamics of realizing collaborations along a value chain, thus, remain underexplored. Therefore, we have only limited insight into how stakeholders engage to reduce perceived ambiguity, understand the implications for their own organizations, and agree on a response. Accordingly, researchers have called for immersive research on the complex social process of circular economy transitions (Bertassini, Ometto, et al., 2021).

By analyzing the interorganizational sensemaking process between stakeholders working to collectively transform a largely linear value chain into a circular one, we provide valuable insights for circular economy researchers and practitioners.

## Method

### Research Setting

Our analysis is based on an in-depth case study of CEFLEX (Circular Economy for FLEXible Packaging), a European industry consortium comprising over 150 companies and representing the entire value chain for flexible packaging, with the mission to transform the value chain from linear to circular. Packaging represents an ideal setting for the study of collaborative efforts toward circular transformation. It is a large established industry that has come under significant public and regulatory pressure due to its current lack of sustainability (European Commission, 2020), prompting an intense search for circular solutions. Given the need for concerted action to realize changes across the value chain, industry stakeholders have started to look for ways to collaborate to formulate collective responses.

The stakeholders of CEFLEX are medium to large companies involved in the production, use, and after-use/recovery and recycling of flexible packaging. CEFLEX is organized into five groups representing the steps of the value chain: (1) material producers, who transform raw inputs such as crude oil, natural gas, or bio-based sources into monomers and polymers, resins, adhesives, inks, coatings, and additives; (2) film producers and flexible packaging converters, who manufacture inputs from material producers into intermediate or final packaging products such as films or foils; (3) brand owners and retailers, who use these inputs to wrap their products and ship them to the point of sale, where they pass to the consumer, who discards the packaging after use; (4) collectors, sorters, and recyclers, who collect, sort, and recycle the discarded packaging to produce input for new (recycled) packaging; and (5) suppliers of sorting and recycling machinery and other industry stakeholders, such as extended producer responsibility associations. As a consortium of industry stakeholders spanning the entire value chain of flexible packaging, it can be considered a specific form of multi-stakeholder initiative that brings together a group of diverse stakeholders with a wide variety of views and interests. CEFLEX’s stated goal is “to make all flexible packaging in Europe circular by 2025” (CEFLEX, 2020, p. 8). Its mission is to increase recycling rates of flexible packaging and, more specifically, achieve the “collection of all flexible packaging and channeling over 80% of the recycled materials into valuable new markets and applications to substitute virgin materials” (CEFLEX, 2020, p. 9). The consortium is governed by a steering committee that includes representatives of all five stakeholder groups.

CEFLEX offers a unique opportunity to study the process of interorganizational sensemaking. The transition toward the circular economy represents a paradigmatic change that challenges the established structures, relationships, and logics of the industry. While packaging has already come under significant scrutiny, demands for more circularity in other industries are growing too, along with initiatives to foster collaboration. Hence, the insights from the collaborative efforts of CEFLEX will also be valuable for other industries.

### Research Process

We followed an engaged scholarship approach (van de Ven, 2007) to study the social dynamics of the alignment process up close. This approach allowed us to generate practical knowledge for the consortium and to valuably contribute to research on interorganizational sensemaking and the circular economy. As we actively engaged with stakeholders, we separated roles and responsibilities among the research team during data collection and analysis, a strategy akin to an insider-outsider approach (Gioia et al., 2010; Louis & Bartunek, 1992). First, during the data collection, four external facilitators (in addition to the author team) assisted during the workshops. Their personal observations provided important input, but the facilitators were not involved during the analysis or the interpretation of the results. Moreover, while two of the authors assumed an explicit insider role of actively shaping and facilitating the process of developing a common position, another author adopted an outsider role, primarily observing the process and collecting and analyzing relevant data to develop our research contributions. Second, during the data analysis, one author became deeply immersed in the data analysis and actively supported CEFLEX in translating the workshop results in practical knowledge. The other two authors were primarily involved in theorizing on the results and outlining the research contributions. Third, we repeatedly discussed the results, theoretical insights, and practical implications with the external facilitators to validate our collective insights.

Our engagement with CEFLEX started in 2019 with the goal of identifying crucial topics on which consortium stakeholders held diverging opinions. The central element of our research was two 2-day interactive workshops with CEFLEX stakeholders during which they formulated aligned positions for two selected topics.

#### Preparation/Scoping

To identify the two topics for the alignment process, we organized a full-day workshop with 25 consortium stakeholders equally representing the five value-chain groups (VCGs). During the workshop, participants reflected on the current linear and prospective circular value chain for flexible packaging and discussed the required changes and implications for industry players to realize the transformation. Based on this reflection, they identified aspects they considered to be contested among stakeholders, leading to a list of 12 salient topics. When we asked participants to choose two topics, two considerations emerged that shaped the dynamics of the discussion: the difference between the topics with regard to the magnitude of disagreement and differences regarding the consortium’s perceived scope of influence. We evaluated all topics along these two dimensions and, after consultation with the steering committee, selected two topics that differed substantially in both dimensions.

The first topic concerned “material preferences for flexible packaging” and whether the consortium should formulate a preference for mono-material packaging over multi-material packaging. The topic was heavily discussed throughout the industry, and there was a high level of perceived disagreement among CEFLEX stakeholders. Individual stakeholders were considered to have a strong direct influence on this topic. The second topic concerned “collection systems for flexible packaging” and whether the consortium should state a preference for collecting post-consumer flexible packaging in a separate stream or via a mixed collection. Overall, disagreement was less pronounced, and many stakeholders considered their scope of influence rather limited.

Comparing the sensemaking process for these two sharply contrasting topics yielded deeper insights into the sensemaking dynamics. The comparison in terms of degree of disagreement allowed us to reveal potential reasons for different levels of engagement and investigate the influence of perception of relevance. The differentiation according to the perceived scope of influence allowed us to dig deeper into the motivation of stakeholders to adapt to achieve change and whom they considered to be driving the change. Selecting two topics for which the interorganizational sensemaking process differed substantially, thus, allowed us to develop a more robust overarching framework.

#### Central Focus/Topic Workshops

As the central element of our research, we organized two 2-day workshops for each of the two selected topics, attended by 25 CEFLEX stakeholders equally representing the five VCGs. During each workshop, we facilitated the negotiation of a joint position statement on the specific topic. We prepared with extensive desk research and six to nine preliminary interviews with stakeholders and industry experts (see Appendix A). We kicked off both workshops with a short presentation on the topic and then broke into five small groups, each including one participant from each VCG. Supported by a facilitator, the participants reflected on the topic, discussed open questions, and negotiated a draft position statement. At the end of the day, we consolidated the five drafts. On the second day, the stakeholders negotiated each element of the new draft position statement in plenum until they reached an agreement. After the workshop, we submitted the position statements to the steering committee who presented them at the general meeting of all stakeholders and subsequently published them (see Appendix C).

#### Data Analysis and Theory Building

Throughout the groundwork and intervention phases, we compiled a rich database including interview data, archival material (confidential documents from CEFLEX, corporate presentations, press statements, etc.), workshop documentation, video and audio recordings, and personal notes. Table 1 provides an overview.

**Table 1.** List of Data Sources.

Data source	Type	Amount (scoping)	Amount (Topic 1)	Amount (Topic 2)	Duration/No. of Pages
Interviews	Semi-structured interviews with workshop participants and other CEFLEX stakeholders	5	6	9	Ø 51 min per interview
Session recordings	Audio/video recordings of workshops (1.5 days per workshop, plenary, and 2*5 parallel breakout groups per workshop)	7	13	13	Ø 75 min per recording
Archival material	Internal memos, guidelines, meeting minutes, presentations, emails, other material	5	5	4	Ø 15 pages
Observations	Field notes from researchers and facilitators from meetings and workshops	6	6	6	Ø 3 pages
Miscellaneous	Workshop documents, photographs from workshops, flipchart drawings, other	80	40	35	Ø 1 page

We focused our analysis on the two topic workshops. We transcribed and coded the interviews and all individual workshop sessions of the phase in MAXQDA. In the analysis, we followed an iterative approach, going back and forth between our empirical data, our forming interpretations, and the sensemaking literature (Eisenhardt, 1989; Yin, 2009). We analyzed the data in three steps: First, for each topic individually, we started with open coding in MAXQDA to structure the data according to the actors involved, the value-chain steps they belonged to, and the type of arguments used. This helped us to identify interesting dynamics in collaborative sensemaking, and we sketched out our first insights as potential themes for further analysis. Second, we compared the processes for the two topics and identified themes and dynamics that had emerged in each one. This allowed us to identify similarities between the processes and to establish general patterns. We focused on the major emerging themes and connected them to the existing literature to work toward a deeper understanding of the process. In particular, we noted that stakeholders used different referencing frames when trying to understand the implications of arguments during the discussions. This observation drew us to map the sensemaking process across different levels—namely, the organization, the value chain, and the ecosystem. In addition, we observed that in both cases, discussions proceeded in various subprocesses within the overall processes. Based on these insights, we developed a general model of the process, differentiating subprocess steps and levels. Also, we noted that the dynamics of collaborative sensemaking and the engagement of individuals to influence the collective process differed substantially between the two topics. As a third step, we went back to the individual topics and reanalyzed them again based on this emerging multi-layer, multi-step process. We then compared the two cases to work out similarities and differences. This comparison allowed us to analyze the characteristics of the topics and conditions causing the sensemaking process to play out differently. Appendix B provides a list of quotes for each topic. Ultimately, our analysis resulted in a general process model, illustrating how the individual subprocesses evolve within and between the different levels of sensemaking, and an application of this model to the two topics, highlighting the differences in sensemaking dynamics within these process steps. To further validate our findings, we also discussed them with the CEFLEX management.

To limit potential bias due to personal involvement in the alignment process, we used three strategies. First, we triangulated our insights from the workshops with findings from the 20 interviews, our document review, and personal notes. Second, we discussed our observations with the neutral facilitators to ensure a breadth of perceptions. Third, we reflected on our involvement, both individually and as a group.

## Findings

**Figure 1.**
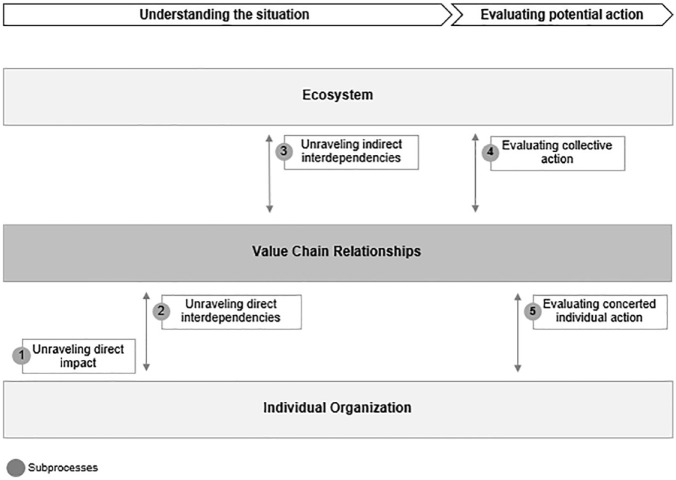
The Interorganizational Sensemaking Process.

Based on our analysis, we developed a general model of the interorganizational sensemaking process, depicted in Figure 1. Within this generalized process, different dynamics of sensemaking unfold, depending on the characteristics of the topics chosen for sensemaking.

Our general model differentiates three levels across which the sensemaking process unfolds: organization, value chain, and ecosystem. The organizational level represents the most granular level of reflection, on which participants seek to understand how the topic and any potential collective position directly affect their own organization. The ecosystem level represents the broadest level of reflection, on which participants make sense of the topic and the implications of potential collective positions for the group of industry stakeholders with respect to its ecosystem. Between these two levels, value-chain relationships represent an intermediate level of reflection, on which participants unravel direct interdependencies between the stakeholders and make sense of their implications. The overall process unfolds across these levels through five subprocesses. The first three subprocesses focus on achieving a holistic understanding of the topic, while the fourth and fifth involve evaluating and aligning on potential actions. Stakeholders go through these subprocesses iteratively, moving back and forth as their understanding broadens until alignment is achieved.

In the first subprocess, participants engage in sensemaking by contextualizing the topic from the perspective of their own organization. The sensemaking is shaped by the participants’ individual knowledge base and organizational identity and reality. Participants are triggered to engage in sensemaking if they perceive the topic to be directly impacting their organization or creating conflict with their evaluation system. Participants evaluate newly acquired information based on its potential immediate implications for their organization and strive for coherence with this evaluation system. These reflections often shape their first opinion and how they initially engage with others.

In the second subprocess, participants make sense of the topic in the context of their relationships with other organizations along the value chain. They reflect on interdependencies between organizations and gain a deeper understanding of how their own organization could be affected by others’ actions. Competing interests can lead to power play between participants. At the same time, knowledge-sharing increases the variety of perspectives and broadens participants’ understanding, raising awareness of the need for alignment.

During the third subprocess, participants unravel interdependencies between the collective industry and its ecosystem. Reflecting on external pressures and industry players’ scope of influence vis-à-vis external stakeholders—especially regulators and consumers—broadens their perspective and highlights potential lack of coherence with established narrow views from lower levels. Industry and group identity play an important role by providing structural context for the process that shapes collective sensemaking—but is also shaped by the process. Increased knowledge and awareness of interdependencies facilitates a broader collective understanding of the topic and greater acceptance for change, thus paving the way for alignment.

During the fourth and fifth subprocess, participants engage to evaluate potential actions and align on an overall collective position. Evaluating the scope of individual and collective actions leads to an agreement on concerted individual or collective action on behalf of the group. These reflections may in turn trigger new iterations of previous subprocesses as the scope of potential actions is connected to the participants’ considerations of identity and common fate. These considerations ultimately lead either to consent with the collective opinion or disidentification.

### Sensemaking for Topic 1: “Material Preferences”

During the first workshop, the central question was whether the consortium should state a preference for mono-material packaging over multi-material packaging. Mono-material packaging contains predominantly one type of material and is, therefore, easier to recycle. In contrast, multi-material packaging blends materials to provide favorable properties such as high product protection and resource efficiency, but its recyclability is limited. While technological innovations might enable the recycling of multi-material packaging in the future, it remains unclear when and indeed whether they might become commercially and ecologically attractive. Material composition plays a crucial role in boosting recycling rates in the short term, which is a central goal of CEFLEX. The participants considered CEFLEX stakeholders to have a strong influence on material choice but held very different views about the right solution.

During the first subprocess, participants initially reflected mainly on the direct implications for their own organizations. They saw the direct relevance of the topic, but while their knowledge was substantial, it was largely limited to their own stage of the value chain. This isolated knowledge shaped a silo mentality and rather narrow sensemaking focused on achieving coherence with their immediate business interest. Accordingly, participants highlighted their own direct interests in the discussion. For example, brand owners argued for a preference for mono-materials to meet consumer demands: “*Some customers are already demanding these things and want to see change*” (brand owner, VCG 3). While multi-material producers highlighted the superior functionality of their solutions, recyclers underlined the need for mono-material input to produce better recyclate.

When the discussion ascended to the value-chain level in the second subprocess, it became highly dynamic as the competing individual interests led to significant power play. Some participants tried to subtly influence the process by seeking to shape the frame of the negotiation: They engaged in framing contests as to what the overarching goal of a circular economy should be and pushed for interpretations that supported their own interests. In particular, while some participants maintained that recyclability should be the key aim, others argued that it should be resource efficiency. While these two goals are connected, privileging one over the other would have different implications for the type of material preferred, and hence for alignment activities. Other participants tried to openly coerce a specific collective outcome. For example, some brand owners threatened to terminate the collaboration altogether and switch to alternative packaging solutions: “*Unless we get some convergence around some sort of standards and focused effort, we are not going to get anywhere. And actually, what that means is we’ll end up with [products] in glass jars*” (brand owner, VCG 3). Given their powerful position, other participants engaged in attempts of sensegiving toward brand owners. For example, multi-material producers addressed them by highlighting the superior importance of product protection: “*You need [multi-materials for their] barrier protection; [. . .] without any kind of protection; you don’t meet [the demands of] your supply chain and generate lots of cost*” (material producer, VCG 1). They underlined how dangerous a switch to mono-materials could be: “*Are you OK with what you’re giving up? [. . .] Are you aware that your customers understand what they have to sacrifice?*” (converter, VCG 2). Still, these endeavors were rather unsuccessful given clear individual interests and a limited willingness to compromise.

At the same time, sensemaking on the value-chain level also raised awareness and understanding of the interdependence between the stakeholders and the resulting need for collective action. Initially, participants had a broad perspective of interdependencies along the value chain. As one participant stated: “*Why are we here? [. . .] Working as individuals doesn’t work, because as individuals you cannot solve it. You should work from an ecosystem perspective, as a value chain*” (workshop participant). However, the process revealed that participants often had a severely limited understanding of how their own actions concretely influenced others and were influenced by them. For example, as a recycler complained, brand owners would always ask for high-quality recyclate for their packaging and blame recyclers for not delivering it but failed to consider that recyclers could only process material that the brands had used in the first place, which often fell short of the quality required. Similarly, multi-material producers and converters offered no insights into how to deal with the associated recycling challenges.

Throughout the process, participants actively engaged to understand the realities of other VCGs, concrete interdependencies, and tradeoffs more deeply. For example, various material producers and converters were grateful to learn about the repercussions of their material choice on the downstream value chain: “*Tell me which direction I need to go, because I will go, and I don’t know*” (material producer, VCG 1). Similarly, while upstream stakeholders were effectively deciding the material composition of the packaging in the market, they acknowledged their dependence on brands: “*It’s up to the brand owner to determine what we need our packaging to do*” (converter, VCG 2). Brand owners, in turn, sought guidance from downstream participants:

*We used to be the ones deciding on the material. Now we need you [recyclers] to tell us what the restrictions are. [. . .] Before, we were the ones asking for what we want; now we need to design for you, basically [. . .] We are looking for guidance from you.* (brand owner, VCG 3)

In particular, participants listened attentively to recyclers—previously considered powerless—as they spoke of the challenges posed by different materials. Yet, despite a clearer understanding of the conflicts and interdependencies, their willingness to compromise was initially low.

In the third subprocess, focused on sensemaking on the ecosystem level, many participants initially found it hard to understand how their organization could be indirectly affected by changes in the ecosystem. They argued for technological solutions on the organizational level and seldom referred to the ecosystem level, if at all. However, some actors actively engaged to challenge this narrow perspective. They highlighted the external threat of potential ecosystem changes and associated repercussions for all stakeholders and actively engaged with other participants to break their sense around individual-level solutions. As one argued: “*There is no time to wait for new technologies. Time has run out*” (recycler, VCG 4). In particular, they highlighted the lack of coherence between the prevailing individual positions and the looming regulatory threat and pushed for better awareness of the need for change to induce a seekership for alternative interpretation schemata. As one participant stressed: “*If we have this discussion and keep all the options open, flexible packaging will just be killed by the legislator*” (workshop participant). That is, they invoked the higher-level goal—to secure the continued existence of the industry—to strengthen a sense of common fate among participants and persuade them to compromise their individual positions.

Yet, many participants had only a limited perception of being part of one industry grouped around a circular value chain and hence of indirect interdependencies associated with being part of this particular group. Other participants, therefore, also engaged in sensegiving to shape the perception of a common industry identity. To encourage group identification, they also used CEFLEX as a reference frame. The association held significant legitimacy due to its representation of leading companies from all steps of the value chain. As one participant reasoned, “*Why do we believe that CEFLEX and the members of the workshop have the ability to take a position? Because we come from a diverse background; we come from experience*” (workshop participant). The participants leveraged the systemic power of CEFLEX as structural context for the debate by pushing for an understanding of the association as a “coalition of the willing” (workshop participant) that wants ambitious concerted action. In effect, some participants who would suffer heavily from a preference for mono-materials came to support such a position as they reflected on the regulatory threat and the need for preemptive action. As a converter working with multi-materials acknowledged, “*If we keep all doors open, which would be the best and easiest for [my company], we will have no credibility, and the politicians will just make the decision for us*” (converter, VCG 2). Contrasting potential direct implications on the organizational level with indirect implications on the ecosystem level ultimately increased their willingness to compromise and paved the way for alignment on concerted action. Ultimately, the negotiations led to a majority of participants favoring mono-material solutions.

During the fourth and fifth subprocess, participants evaluated potential actions and agreed that a shift to mono-materials could be achieved through concerted organization-level changes. Hence, they saw the position statement as a guide to harmonize individual actions. The resulting statement voiced a clear preference for mono-materials. It was relatively ambitious and included a direct call to action to all CEFLEX stakeholders to revise their activities to align with this preference. The consortium leader presented the position statement at the following general meeting of CEFLEX stakeholders, and it was published 8 months later (see Appendix C). However, some disidentification also occurred, as some participants rejected the newly formed collective sense. Upon publication, four participants who had opposed the preference for mono-materials voiced their criticism in an open letter and sought to reopen the discussion—but did not succeed.

### Sensemaking for Topic 2: “Collection Systems”

The second workshop focused on the question of whether CEFLEX should state a preference for collecting post-consumer flexible packaging in a separate stream or via a mixed collection with residual waste. For CEFLEX to reach its objective of all flexible packaging being collected, the prevailing rate of collection needs to be significantly increased. While most flexible packaging is collected separately (along with other packaging) on the household level in the European Union (EU), a significant portion ends up in the residual waste and is not recycled. To increase the collection rate, alternative routes are available, from actions to improve separation at household level to switching to alternative systems such as a mixed collection of (flexible) packaging together with other waste streams and later separation at industrial sites. Examples of such post-sorting exist but are mostly in the pilot phase. Overall, the participants’ knowledge about the topic was very limited. As waste collection is regulated by local authorities in the EU, stakeholders perceived the consortium’s influence as rather limited. There was some perceived divergence of opinion among stakeholders, but overall, disagreement was less pronounced.

As for the first subprocess, participants initially engaged in very little sensemaking on the organizational level. Knowledge and even awareness of the topic and the challenges of the current collection system were very limited, apart from a few participants who were directly involved in alternative collection pilots. Much of the value chain felt completely detached from the task of collection:

*Maybe in the first position workshop there were more people [. . .] who would be directly impacted by the outcome of the position statement. Whereas here [. . .] we do have a vested interest because we are all part of this process and the value chain, but no one’s direct business is going to have to start processing these materials. I think this slightly changes how we approach the discussion.* (consortium manager)

Accordingly, there was no feeling of conflict, doubt, or void that would induce a search for sense. Indeed, many participants refrained from voicing any opinion at all. As one material producer admitted, “*I suppose the different sorting and recycling companies are more expert in this than I am, because I am a producer*” (material producer, VCG 1). To create relevance for the sensemaking process, many devolved to relating to the topic personally, as consumers. For example, one participant pointed out how consumers might find the current collection system confusing:

*If I imagine my grandma is sitting at home and has to work out: Is this plastics, paper, or glass? Or this plastic with the cheese inside, should I put it here or put it there?* (machine producer, VCG 5)

Equally, during the second sensemaking subprocess on the value-chain level, participants initially saw no interdependencies with their direct business partners either. Consequently, they felt little conflict and little need to engage in sensemaking. As one recycler complained, “*The rest of the value chain has no insight. It seems that they all got too comfortable with the existing system*” (recycler, VCG 4).

Also, power play was limited as no individual actor was perceived to have direct power to coerce a decision. The few participants active on the topic tried to engage in sensegiving by highlighting the limitations of the current system, but many others suspected their motives: “*Everybody has their own interests. [. . .] So, it’s difficult to get a good view of the real best practices*” (consortium manager). Some participants even openly accused the active participants of being self-interested, as their “*economic interest in the position [was] crystal clear*” (workshop participant, VCG 5). That is, because many participants felt little need for sensemaking, they mistrusted those who were pushing for new sense. This skepticism hampered subsequent openness and learning.

In addition, many shared the perception that collection was primarily a regulatory issue, as the decisions were made by stakeholders outside the industry—namely, public-sector authorities: “*This is not down to goodwill. This is going to be set up by law*” (workshop participant, VCG 5). Hence, it was not the dependence among stakeholders along the value chain that mattered but their collective dependence on the regulator. This perceived inability to influence the subject led to indifference about making sense of it.

Sensemaking only took off during the third subprocess, when a small group intensively pushed to bring the debate to the ecosystem level. They actively strengthened this broader reference frame to underline that the topic was highly relevant for all actors—albeit indirectly. To achieve this, they particularly engaged to shape the perception of a group identity. In particular, they pushed for awareness of collective interdependence vis-à-vis a massive regulatory threat and the relevance of collection for all stakeholders. By highlighting this common fate, they sought to overcome the prevailing mistrust and instill a sense of ownership of the challenge facing the industry. As one argued, “*It is the industry’s responsibility to design a well-functioning collection system*” (workshop participant).

These discussions triggered a reevaluation of identity and a sense of belonging that helped to induce the seekership required to engage in sensemaking. Over time, the other participants developed some connection to the topic, and the discussion strengthened the perception of indirect interdependence: “*We should emphasize that the public is not responsible for a system that doesn’t work. And that we [the industry] should take responsibility*” (workshop participant).

However, actors were still unsure as to how they could influence this topic. In line with the consumer perspective, many participants considered the main lever to be better consumer education within the existing system, without any consideration of change in the industry. Yet, statements were often emotional or normative rather than factual: “*Everybody should be part of collection. Consumers should not be part of the problem, but part of the solution*” (workshop participant). In a sense, they externalized the problem by pointing toward improvements outside their scope of influence.

The limited perceived scope of influence was also connected to the widespread perception of CEFLEX’s identity as a “*technical exchange platform*” (workshop participant). Because many participants saw the consortium’s role as organizing technical pilots and sharing data, they were slow to see how it could act on this topic. During the process, a subgroup of participants deliberately engaged to broaden this perceived scope of influence by introducing an alternative role for CEFLEX. They highlighted the possibility that CEFLEX could engage as an advocacy body in the political realm and use its voice to change the conditions in the ecosystem set by legislators. Reshaping the consortium’s identity, in turn, altered what participants considered to be legitimate activities of the group. This opened up the possibility to at least induce change indirectly, which helped hesitant participants relate to the topic. Thus, the consortium itself became an important reference frame for the sensemaking process. While many participants remained somewhat skeptical about alternative collection options to the last, the subgroup managed to enhance the relevance of the topic and, importantly, evoke a feeling of empowerment by underlining a potential active role.

Given the considerations for political engagement, the evaluation of potential action during subprocesses four and five focused on collective actions on the ecosystem level. In line with the advocacy identity of CEFLEX, participants brought up the idea of publishing a statement, addressed mainly toward stakeholders external to the industry, to promote a change in the current legislative system. Interestingly, the consortium leader himself engaged heavily in this subprocess. On several occasions, he directly intervened in the discussion to stress the need to formulate a political position that would achieve systemic change: “*What we are talking about here is a policy statement that acts as a compass, that sets a direction of where we want go*” (consortium leader). Most current regulations would state a preference for separate collection, while other options needed to be considered: “*The way the position statement needs to finish today is to open the door to post-sorting of mixed waste collection—because that door is currently firmly shut*” (consortium leader). Due to the leader’s authority as an independent manager, participants did not question his opinion as they had with those individual participants who had voiced a similar preference. This led to broader acceptance of a political statement and to CEFLEX advocating the investigation of alternative collection systems.

Toward the end of the negotiation, a consensus evolved around the need to raise awareness of insufficient collection rates and explore alternative collection options. The final position statement was careful to explain why CEFLEX was issuing a position statement on the topic in the first place and was generally cautious, arguing for a general preference for separate collection corresponding to most current legislation, but opening the door for additional alternative options. The steering committee presented the position statement at the subsequent general meeting and published it 7 months later (see Appendix C).

### Cross-Case Comparison

**Figure 2.**
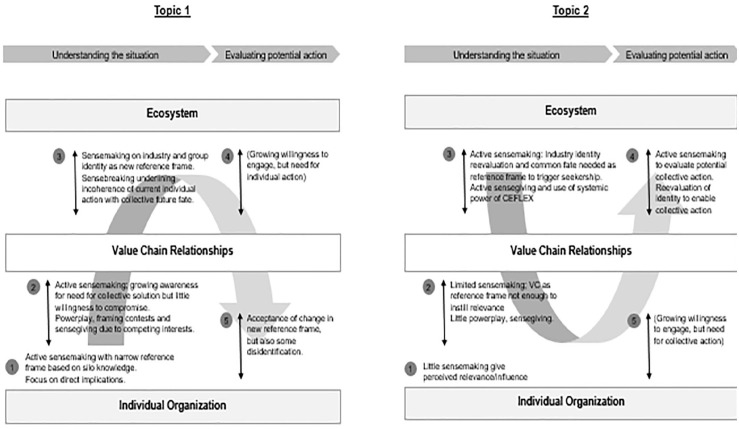
Comparison of Process Dynamics Between Cases.

In both cases, the negotiations resulted in a position statement backed by the majority of participants. While the cases show similarities in the overarching structure of sensemaking, interesting differences can also be observed. Comparing the two allows us to elaborate on important aspects of sensemaking within our general model, as illustrated in Figure 2.

In both cases, interorganizational sensemaking unfolded across the organizational, value-chain, and ecosystem levels. Sensemaking on each level was central to the overall success of the process, as each one provided a different context for the sensemaking.

However, important differences in the dynamics of the process can be observed. In particular, we saw that the processes unfolded differently across these levels depending on the participants’ consideration of direct impact and influence. While both topics had been collectively chosen for their overarching relevance, when it came to the actual sensemaking process, participants differed over whether they perceived it to be relevant to their companies.

For Topic 1 on material preferences, participants mostly considered the topic to be directly influential for their organizations. They were eager to engage in the interorganizational process, as they had a direct interest in understanding the topic. Therefore, sensemaking started strongly on the organizational level. Yet, this process suffered from silo knowledge and unawareness of greater interdependencies. Thus, the collective process moved up to the second and third levels to allow for broader systemic context. Sensemaking around value-chain relationships was important in helping participants understand the interdependencies between their activities. But only sensemaking on the ecosystem level and the acceptance of a common fate led to a willingness to compromise. This ultimately led to alignment for joint action on the organizational level, as participants traded off their individual short-term interests for the sake of a collective goal. That is, the overall process started and ended with strong engagement on the organizational level but required intermittent engagement on the value-chain and ecosystem levels to create an understanding of interdependencies and induce a willingness to compromise.

For Topic 2 on collection systems, most participants perceived no direct impact or influence, so sensemaking was initially almost negligible at the organization and even value-chain levels. True sensemaking only really started on the ecosystem level when the process created first-time awareness of this largely neglected topic. The reflection of the collective interdependence of the group vis-à-vis external stakeholders in the ecosystem in turn induced a sense of relevance and seekership that triggered sensemaking of the repercussions of this topic on the value-chain level and, ultimately, the organizational level too. This played an important role in creating an understanding of the need to act. The actual search for potential joint action then played out again on the ecosystem level, as little could be done on the organizational level. However, defining potential action also required changing the perceived identity of the group. Only the acceptance of a potential advocacy role for the consortium revealed a way to actually influence the topic and ultimately led to an agreement for collective political action.

## Discussion

Our analysis of how stakeholders of a value-chain consortium engage to jointly make sense of the transition from a linear to a circular value chain contributes to both the sensemaking and circular economy literatures and provides practical recommendations.

### Contributions to Research on Sensemaking

We provide three main insights into the process dynamics of interorganizational sensemaking.

First, we show how interorganizational sensemaking between interdependent actors evolves across different levels: organization, value chain, and ecosystem. So far, research has primarily suggested that influences from industry on the organizational level matter for the sensemaking process (Cristofaro, 2022; Patvardhan et al., 2015; Stigliani & Elsbach, 2018). Our findings reveal the dynamic interplay of influences and the unfolding of the sensemaking process across three levels. On the organizational level, actors make sense of the direct implications of the issue on their own organizations. On the value-chain level, sensemaking helps them understand the implications of direct interdependencies with others. On the ecosystem level, sensemaking reveals the implications of indirect interdependencies. Engaging on all levels is important for the overall process as it serves several interconnected purposes: (a) Engaging on different levels helps create the relevance and seekership necessary for actors to engage in the process. Given the efforts associated with sensemaking, actors will only engage in it if they consider the issue to be relevant to their organization (Maitlis & Christianson, 2014; Pratt, 2000). Stakeholders initially evaluate the relevance of an issue based on a narrow organizational perspective. Engagement on the value-chain and ecosystem levels helps organizations understand how they may be affected by belonging to a certain industry and through its relationships with other external stakeholders, which strengthens their perception of the topic’s relevance for them. (b) While Seidl and Werle (2018) highlight the value of a breadth of perspectives in interorganizational sensemaking, our research shows that engaging on the different levels also contributes a depth of perspectives that enables individuals to gain a holistic understanding of the topic. This holistic understanding helps stakeholders go beyond technical knowledge and consider interorganizational dynamics of different interests, trade-offs, and power plays, as well as the potential repercussions of interdependencies for their own organizations. (c) The reflection across levels not only creates an understanding of the topic but, importantly, also helps to instill a willingness to compromise, which is a prerequisite for alignment. In particular, an understanding of direct and indirect interdependencies is essential for making participants aware of their own interdependencies and prepared to accept a common fate, which ultimately leads them to embrace necessary trade-offs.

Second, we extend Schildt’s (2020) work on power in sensemaking by providing insights into the dynamics of episodic and systemic power across the different levels of sensemaking. Our findings indicate that systemic power is relevant across all levels but plays out differently on each one, as the different levels can also be considered different reference frames or systemic contexts for the collective sensemaking process (Kaplan, 2008; Schildt et al., 2020). As the context of sensemaking changes across the levels, so does the systemic power imposed by these structural contexts on the sensemaking process. On the organizational level, the narrow context often induces conservative influences leading to committed narrow forms of sensemaking to preserve coherence with direct organizational interests. Instead, the broader reference frames on the value-chain and ecosystem levels have more reformative influences on the sensemaking process as they embrace alternative concepts and new evaluation criteria. Episodic power is used especially on the value-chain level, where direct interdependencies and direct divergent interests most prominently clash. Episodic power is often used to reinforce the status quo and established beliefs. But it can also be used in inspirational and expansive manner, to problematize established goals and provide actors with new observations and ideas that induce sensemaking on different levels and, thus, within different reference frames.

Third, our findings extend research on the influence of identity in the sensemaking process. Research on industry identity formation has thus far alluded to an interplay between perceptions of identity on the organizational and industry levels (Patvardhan et al., 2015; Stigliani & Elsbach, 2018). We shed further light on such interplay in the context of interorganizational sensemaking. In particular, our findings indicate that external shocks may challenge an established industry identity and that the reconceptualization of that identity both influences and is influenced by the collective sensemaking process. Interorganizational sensemaking may be needed to jointly reestablish a shared industry identity in the light of a changing environment. In addition, this reconceptualized industry identity influences interorganizational sensemaking, as it provides a new interpretative frame for the implications of the ecological shock on the ecosystem level and indirectly for the participating organizations. Such a reciprocal interplay underlines the importance of achieving a coherent perception of industry identity shared by all participating organizations for a successful collective action as an important part of the interorganizational sensemaking process.

Ultimately, aligning on a joint pathway depends on establishing a collective identity, as this is needed for the notion of a common fate to be accepted and, thus, for willingness to compromise to be instilled. Such collective identity may also be facilitated through the group structure of a stakeholder initiative, such as an industry consortium. The collectively accepted identity of a particular group or initiative may function as a conducive reference frame for the ecosystem-level sensemaking that might be more relatable for participants than the more diffuse notion of industry identity. Resonating with Lawrence et al. (2012), such a group identity may become a part of the reference frame for sensemaking whose systemic power then helps to institutionalize and legitimize change.

### Contributions to Research on the Circular Economy

Our study also contributes to the circular economy literature. While previous research has frequently called for collaboration to realize the transition to a circular economy in general and has readily pointed to the interdependencies between actors along value chains, little research so far addresses the challenges of such collaborations or suggests how an alignment between interdependent actors with diverging interests can actually be achieved (Brown et al., 2021; Centobelli et al., 2020; Eikelenboom & de Jong, 2022; Pieroni et al., 2019). We respond to calls for more research on the social processes of the collaborative transition to the circular economy by providing insights into how such collaborative initiatives may be successfully realized (Bertassini, Ometto, et al., 2021; Brown et al., 2021; Parida et al., 2019; Pieroni et al., 2019).

Our findings suggest that an alignment between interdependent stakeholders along a particular value chain requires a joint reflective process across the organization, value-chain, and ecosystem levels. The joint reflection across these levels is essential for creating an agreed-upon understanding of the transition. Given the lack of a common understanding of the circular economy concept and the resulting uncertainty (Kirchherr et al., 2017; Korhonen et al., 2018; Moraga et al., 2019), the collaborative process is not only important for bringing together isolated knowledge on the organizational level but also for connecting the different realities on the value-chain level and for creating awareness about ecosystem-level influences. It also facilitates achieving a joint understanding of the overarching goal for the respective industry and a joint vision of the circular value chain. Connecting the organizational-level implications of individual actors with the direct interdependencies on the value-chain level and the indirect interdependencies on the ecosystem level is essential for achieving alignment on a particular way forward.

Researchers have thus far highlighted the existence of interdependencies and diverging opinions, trade-offs, and power plays among actors (Bening et al., 2021; Kirchherr et al., 2018; Tapaninaho & Heikkinen, 2022). Our analysis suggests that a collaborative assessment of the direct and indirect interdependencies between organizations allows individual stakeholders to connect the direct implications for their organization with the ones resulting from these interdependencies and, thus, to create an understanding of the trade-offs. This reflection strengthens the sense of a common interconnected fate among stakeholders and of belonging to and identification with an industry grouped around a circular value chain. It also forges a willingness to compromise individual short-term goals, without which alignment between the diverse actors cannot be achieved.

### Managerial Implications

Our work also offers valuable insights for the management of diverse stakeholder alliances.

First, the success of collaborative efforts that span value chains may strongly depend on the selection of participants. Including participants from the entire value chain is important for the transition toward a circular economy. However, our findings suggest that direct and indirect dependencies may play out differently depending on the specific topic to be tackled and that stakeholders contribute differently to the alignment process. Some stakeholders are already active and understand the relevance of the given topic, while others appear more passive and distant from the topic. Thus, for achieving an actionable alignment among actors, managers of diverse stakeholder alliances should carefully curate the list of participants and may have to actively reach out to more passive but relevant stakeholders.

Second, our findings show that participants engage differently in the five subprocesses depending on the nature of the topic. For example, during the alignment process on collection systems, sensemaking efforts on the organizational and value-chain levels were initially rather unproductive and cumbersome, as stakeholders lacked any connection to the topic. Collective sensemaking only really kicked off at the ecosystem level once participants developed a sense of relevance, which happened when they became aware of indirect interdependencies within this broader reference frame. This relevance, in turn, also triggered active engagement on the lower levels. Thus, practitioners facilitating similar alignment processes should ensure that the focus and sequence of subprocesses are tailored to the characteristics of the chosen topics.

Third, the organizational body of stakeholder initiatives plays a conducive role in the overall alignment process. Often, such bodies enjoy considerable legitimacy and authority among participants, and their representatives can provide impartial input to the negotiation. In addition, as our research indicates, the organization itself may also strengthen an ecosystem-level reference frame that helps participants better understand indirect interdependencies and consider a broader scope of potential action. Facilitators can also leverage the power and legitimacy of the organization to strengthen a group identity—not only to attract the right participants but also to steer a progressive process.

### Limitations and Future Research

Our research has several limitations that indicate opportunities for future research.

First, our research focuses on a facilitated sensemaking process over a specific period. Yet, sensemaking is a dynamic concept, with the sense made connected to a temporary perception of an issue or event. In addition, the issue or event to be made sense of might be further developing over time—like the continuously worsening climate crisis, for instance. Future research that examines the temporal aspects of interorganizational sensemaking may develop important insights into the regularity and time-sensitivity of interorganizational sensemaking.

Second, our study is based on the collaborative efforts of a single value-chain consortium in the packaging industry, and we have only studied the collaborative efforts for two selected topics. The dynamics of direct and indirect interdependencies among actors of one value chain and requirements for concerted action to close resource loops are likely similar in other industries. Nevertheless, some of the observed challenges and dynamics might be idiosyncratic to the flexible packaging value chain, this particular consortium, or the two topics we examined. Future research in different industries or topics may clarify the transferability of our findings to other research settings.

Third, as we sought to develop insights into the dynamic process of interorganizational sensemaking, we benefited extensively from our active involvement with the consortium. Yet, such an engaged scholarship approach (van de Ven, 2007) also bears the risk of bias in the collection and interpretation of data. Although we have taken various steps to ensure the validity of our results, some minor bias cannot be conclusively eliminated. Given these limitations, future studies that analyze the alignment work of other stakeholder collaborations and systematically compare such efforts may be useful to further corroborate and develop our findings.

## Conclusion

We have analyzed the interorganizational sensemaking process between stakeholders of a consortium from the flexible packaging industry seeking to transition from a linear to a circular value. We observed that interorganizational sensemaking unfolds in various dynamic and interconnected subprocesses on the organizational, value-chain, and ecosystem levels. The interplay of these processes helps participants broaden their perspective and embrace the implications of direct and indirect interdependencies between stakeholders. These different considerations ultimately induce a willingness to compromise and open the door for collective alignment on joint action. Our research contributes to the sensemaking literature by shedding light on the process of interorganizational sensemaking of interdependent actors and provides insights into stakeholder collaborations promoting the transition to a circular economy.

## Figures and Tables

**Figure:**
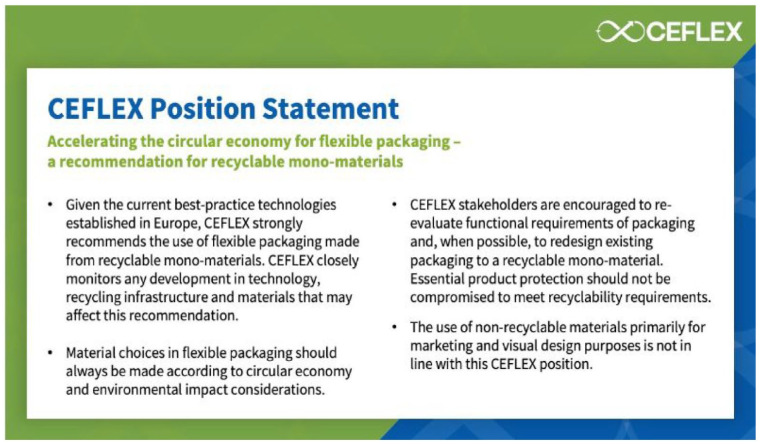
Position statement for Topic 1 “Material Preferences for Flexible Packaging”. https://ceflex.eu/first-position-paper-points-to-collaborative-effort-towards-a-circular-economy/

**Figure:**
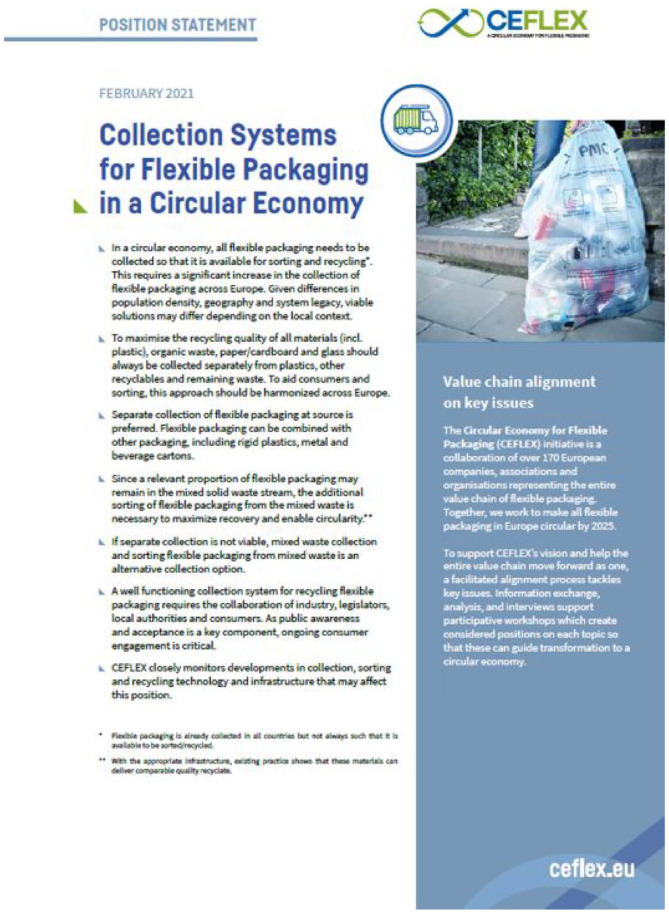
Position statement for Topic 2 “Collection Systems for Flexible Packaging”. https://ceflex.eu/position-paper-collection-systems-for-flexible-packaging-in-a-circular-economy/

## Appendix A

**Table:** List of Interviews.

No.	Value-chain step	CEFLEX/external	Groundwork	Prior workshop 1	Prior workshop 2	Duration	Date
1	Material producers (1)	VC group leader	x			60 min	September 18, 2019
2	Material producers (1)	VC group leader		x		55 min	January 15, 2020
3	Material producers (1)	VC group leader			x	40 min	March 06, 2020
4	Film producers and converters (2)	VC group leader	x			50 min	September 23, 2019
5	Film producers and converters (2)	VC group leader		x		45 min	January 21, 2020
6	Brands and retailers (3)	VC group leader	x			50 min	September 13, 2019
7	Brands and retailers (3)	VC group leader		x		30 min	January 13, 2020
8	Sorters, collectors, and recyclers (4)	VC group leader	x			70 min	September 21, 2019
9	Sorters, collectors, and recyclers (4)	VC group leader		x		60 min	January 16, 2020
10	Sorters, collectors, and recyclers (4)	VC group leader			x	70 min	February 26, 2020
11	Sorters, collectors, and recyclers (4)	External expert		x		45 min	January 13, 2020
12	Suppliers, end users, and others (5)	VC group leader	x			50 min	September 21, 2019
13	Suppliers, end users, and others (5)	VC group leader		x		35 min	January 14, 2020
14	Suppliers, end users, and others (5)	VC group leader			x	40 min	February 28, 2020
15	Suppliers, end users, and others (5)	CEFLEX stakeholder			x	55 min	February 26, 2020
16	Suppliers, end users, and others (5)	CEFLEX stakeholder			x	45 min	March 06, 2020
17	Sorters, collectors, and recyclers (4)	CEFLEX stakeholder			x	55 min	March 17, 2020
18	Sorters, collectors, and recyclers (4)	External expert			x	55 min	March 18, 2020
19	Sorters, collectors, and recyclers (4)	External expert			x	60 min	February 25, 2020
20	Sorters, collectors, and recyclers (4)	External expert			x	45 min	February 24, 2020

## Appendix B

**Table:** List of Quotes.

Process step	Topic 1 (mono-material vs. multi-materials)	Topic 2 (collection systems)
Subprocess 1: **unraveling direct impact** (organization)	“*In the end it’s the brand owner who specifies the packaging.*” (brand owner, VCG 3). “*[The topic is] about explaining that packaging has a serious function—to protect, not just contain—and that is not clear to everybody.*” (converter, VCG 2) “*We need [multi-materials for their] barrier protection; [. . .] if you need something protective, it’s not just mono.*” (material producer, VCG 1) “*As recyclers, we have been demanding mono fractions and improved packaging design for a long time. We just can’t influence it.*” (recycler, VCG 4)	**“*I suppose the different sorting and recycling companies are more expert in this than I am because I am a producer. I can talk about this as a consumer*.” (material producer, VCG 1)** **“*Maybe in the first position workshop there were more people [. . .] who would be directly impacted by the outcome of the position statement. Whereas here [. . .] we do have a vested interest because we are all part of this process and the value chain, but no one’s direct business is going to have to start processing these materials. I think this slightly changes how we approach the discussion.*” (consortium manager)** **“*If I imagine my grandma is sitting at home and has to work out, ‘Is this plastics, paper, or glass? Or this plastic with the cheese inside, should I put it here or put it there?’*” (machine producer, VCG 5)** “*I need to tell you; I haven’t thought much about this until now.*” (material producer, VCG 1) “*From my point of view we are still on a journey, and we don’t fully understand what quality is actually needed.*” (brand owner, VCG 3) “*In my opinion, the whole topic of collection is being pushed by [companies involved in trials with alternative collection systems]. But I am interested to see whether there are more stakeholders thinking into this direction.*” (recycler, VCG 4) *[In response to being asked what influence the topic would have on the organization]*: “*I am not sure whether it would have any influence.*” (material producer, VCG 1)
Subprocess 2: **unraveling direct interdependencies** (organization-value chain)	“*I am thrilled that we have now reached a point where the various groups in this value chain are talking to each other—which wasn’t the case in the past—and an understanding of problems across value chains is finding its way into CEFLEX and the economy.*” (recycler, VCG 4) **“*It’s up to the brand owner to determine what we need our packaging to do.*” (converter, VCG 2)** **“*We used to be the ones deciding on the material. Now we need you [recyclers] to tell us what the restrictions are. [. . .] Before, we were the ones asking for what we want; now we need to design for you, basically [. . .] We are looking for guidance from you.*” (brand owner, VCG 3)** **“*You need barrier protection. you can’t say ‘it is not critical’, say ‘just use mono[-material]’ without any kind of protection; you don’t meet [the demands of] your supply chain and generate lots of cost.*” (converter, VCG 2)** “*We need to explain to our customers and the value chain why we should in some cases use multi-materials; why mono-materials are not fitting their needs. This means working with the brand owners and the retailers but also with the producers.*” (converter, VCG 2) “***Are you OK with what you’re giving up [when switching to mono-material]? [. . .] Are you aware that your customers understand what they have to sacrifice?*” (converter, VCG 2)** “*[A clear preference for mono-materials] sounds too revolutionary. The wording is too harsh. [. . .] Brand owners—for them, visual marketing and visual design is a very important sales and marketing tool they can’t live without.*” (material producer, VCG 1) “*It’s about shared responsibility along the value chain.*” (workshop participant)	“*A lot of knowledge is out there, but I don’t believe it is well connected.*” (consortium manager) **“*The rest of the value chain has no insight. It seems that they all got too comfortable with the existing system.*” (recycler, VCG 4)** **“*Everybody has their own interests. [. . .] So, it’s difficult to get a good view of the real best practices.*” (consortium manager)** **“*The economic interest [of machine producers] in the position is crystal clear.*” (workshop participant)** “*As for the brand owners, I would assume that they are not so much involved in the waste issue as to have a well-founded opinion on it.*” (recycler, VCG 4) “*I know from [a brand owner] that they have started to calculate how such [collection] systems work along the whole value chain [. . . .] and they have started to ask: Can we as [a brand] not influence these structures?*” (recycler, VCG 4)
Subprocess 3: **unraveling indirect interdependencies** (value chain-ecosystem)	**“*There is no time to wait for new technologies. Time has run out*.” (recycler, VCG 4)** “*If we don’t do anything, we will lose our license to operate.*” (workshop participant) **“*If we keep all doors open, which would be the best and easiest for [my company], we will have no credibility, and the politicians will just make the decision for us.*” (converter, VCG 2)** **“*Some customers are already demanding these things and want to see change.*” (brand owner, VCG 3)** “*The regulator becomes more and more uncontrollable because the regulator feels the pressure from the street.*” (recycler, VCG 4) “*What is [the regulator’s] priority? It’s appealing to their electorate.*” (recycler, VCG 4) **“*If we have this discussion and keep all the options open, flexible packaging will just be killed by the legislator.*” (workshop participant)** **“*Why are we here? [. . .] Working as individuals doesn’t work, because as individuals you cannot solve it. You should work from an ecosystem perspective.*” (workshop participant)** “*We can’t bet everything on the future.*” (brand owner, VCG 3) “*[Some stakeholders] keep abusing CEFLEX as an alibi event, following the motto ‘Let’s talk a little about packaging design, and then we’ve done our duty, and the world will be a better place.’ [. . .] I have the impression that several participants do not want to see that we need a paradigm shift here.*” (recycler, VCG 4)	**“*This is not down to goodwill. This is going to be set up by law.*” (workshop participant, VCG 5)** “*When talking about collection, there is a huge influence of the first part of the chain which is consumers.*” (converter, VCG 2) “*As an industry, we are entering an area which is not our home turf. We are entering the discussion with municipalities.*” (brand owner, VCG 3) “*If I was a brand owner, I would say, we are doing a lot of effort to make the plastics packaging circular and we are doing it because if not, we feel we are going to lose our consumers (. . .). So, this is because the public reaction to the perceived lack of circularity of plastics packaging is at the source of all the efforts we are making.*” (workshop participant) “*Even after 30 years of separate collection, we see that citizens do not always do what they are asked to do. That’s a reality we need to take into perspective also for our future planning.*” (workshop participant)
Subprocess 4: **evaluating collective action** (value chain-ecosystem)	“*We said we are not [an] advocacy [body] so we cannot use words like ‘oppose’ because that is advocacy.*” (workshop participant) “*A key pillar of the work of CEFLEX is to work on advanced recycling solutions also for multi-materials. (. . .) If we focus here only on existing technologies for mono-materials then there is no future-oriented perspective.*” (material producer, VCG 1) “*If you want to actively address this issue, then you should indeed make political statements now as a signal to society and a signal to the regulator.*” (recycler, VCG 4) “*We are at the beginning of a long journey. It requires to completely redesign 20 years of packaging solutions. And in CEFLEX, with the power we have in terms of R&D and research and influence, we need to focus all the power and money here.*” (workshop participant) “*We want to be a technical information platform.*” (workshop participant)	**“*Everybody should be part of collection. Consumers should not be part of the problem, but part of the solution.*” (workshop participant)** “*If we just send the message to consumers that it is fine to stick it all in one bin and we will sort it out for you, it doesn’t place any kind of ownership on them.*” (brand owner, VCG 3) “*I see much too much focus on this stupid customer who doesn’t do the right thing and I totally disagree with that. [. . .] The system should be so simple that you can hardly make any mistakes. If you are relying on the consumer, then we are doing the wrong things.*” (workshop participant) **“*We should emphasize that the public is not responsible for a system that doesn’t work. And that we [the industry] should take responsibility.*” (workshop participant)** **“*It is the industry’s responsibility to design a well-functioning collection system.*” (workshop participant)** “*If CEFLEX just puts out you just have to sort the separately collected and everything is fine and then the quotes will not be achieved because everyone is relying on that, then the public outcry might be just as big as it is right now, and I want to reduce that as fast as possible.*” (machine producer, VCG 5) “*The way the position statement needs to finish today is to open the door to post sorting of mixed waste collection because that door is currently firmly shut. The way I would like the position statement to end up is to say: ‘We prefer separate sorting, we need it (. . .) but we acknowledge that sometimes it needs alternative solutions.’.*” (consortium leader) “*We want to do as much segregated separation as possible and only where that is practically impossible, would we do post[-sorting]. And I am saying that as an organization who is actually picking up the bill for this by the way, so I think we need to be clear about who is paying for what.*” (brand owner, VCG 3) “*It is not a politically correct thing to say that you are going to need to use post-sorted materials as a collection system, but it is increasingly a reality in certain situations.*” (consortium leader)
Subprocess 5: **evaluating concerted individual action** (organization-value chain)	“*CEFLEX is not an industry association. It’s a* ***coalition of the willing***. *And anyone who is not happy with that should get out.*” (workshop participant) “*It’s about guidelines, the star on the horizon, and chasing that and giving the inspiration to R&D to work for the solutions.*” (material producer, VCG 1) **“*Unless we get some convergence around some sort of standards and focused effort, we are not going to get anywhere. And actually, what that means is we’ll end up with [products] in glass jars.*” (brand owner, VCG 3)** “*I want to go, because my company wants to help the world, sustainability. . .* ***Tell me which direction I need to go, because I will go, and I don’t know***.” (material producer, VCG 1) **“*Why do we believe that CEFLEX and the members of the workshop have the ability to take a position? Because we come from a diverse background; we come from experience.*” (workshop participant)** “*Mechanical recycling is currently the only industrially viable way to do recycling. Because it is urgent, we have to develop that, and we need the recyclable mono-materials to increase the yield.*” (material producer, VCG 1)	“*What we are talking about here is a policy statement that kind of acts as a compass, that sets a direction of where we want go.*” (consortium leader) “*Having a CEFLEX position gives an industry agreed position. So, we are all coming from the same angle, the same point of view, rather than being disjointed.*” (workshop participant) “*We are talking about huge investments, and we need to have a long-term plan for the companies of the whole value chain how to work with this and where are we going.*” (workshop participant)

*Note.* VCG = value-chain group; R&D = Research & Development.
